# Influence of aging, macronutrient composition and time-restricted feeding on the rat gut microbiome

**DOI:** 10.1093/geroni/igaa057.3278

**Published:** 2020-12-16

**Authors:** Abbi Hernandez, Sara Burke, Thomas Buford, Christy Carter

**Affiliations:** 1 University of Alabama at Birmingham, Birmingham, Alabama, United States; 2 University of Florida, Gainesville, Florida, United States

## Abstract

Declining health and cognition are hallmarks of advanced age that reduce both the quality and length of the lifespan. While caloric restriction has been highlighted as a strategy for increasing healthspan, time-restricted feeding and changes in dietary macronutrient composition may be more feasible alternatives with similar health outcomes. Furthermore, age-related changes in gut microbiome composition may reciprocally interact with several physiological systems – providing a good target for future therapeutic interventions. To begin to investigate the potential utility of a ketogenic (high fat, low carbohydrate) diet and/or time-restricted feeding, fully mature young (5 mo) and older (22 mo) adult male Fischer Brown Norway Hybrid rats were placed on a time-restricted feeding regimen of a ketogenic or micronutrient and calorically matched control diet for 7 months. A third group of rats was permitted to eat standard chow ad libitum. Fecal samples collected at the conclusion of the study were submitted for 16S microbiome analysis, which revealed significant differences across age and diet groups, as well as across feeding paradigms. Beta diversity analysis demonstrated distinct microbiome composition across the three diet groups regardless of age. Furthermore, diet group significantly impacted abundance in expression of several microbiota at the phylum level, including Verrucomicrobia, Cyanobacteria, Actinobacteria and Patescibacteria, though age did not. Verrucomicrobia was significantly increased (p=0.02) and Actinobacteria and Patescibacteria (p<0.01) were significantly decreased in animals fed in a time-restricted fashion. These results indicate the value of both altered macronutrient composition and altered feeding methodology for therapeutic interventions targeting the gut microbiome.

